# Elastic Intramedullary Nailing of a Medial Clavicle Fracture in a Pediatric Patient

**DOI:** 10.1155/2017/6354284

**Published:** 2017-05-16

**Authors:** Michael J. Stark, Michael J. DeFranco

**Affiliations:** Nova Southeastern University College of Osteopathic Medicine, 3301 College Avenue, Davie, FL 33314, USA

## Abstract

**Introduction:**

Injuries to the medial clavicle in pediatric patients typically involve the physis and/or sternoclavicular joint. Clavicle fractures are one of the most common injuries in children, but ones at its medial end are rare. Most medial clavicle fractures are treated nonoperatively, but surgery is indicated in some cases. This original case report is unique in describing the use of an elastic intramedullary nail for fixation of a completely displaced medial clavicle fracture in a pediatric patient.

**Case Presentation:**

A pediatric patient sustained a completely displaced fracture of the medial clavicle. The fracture was lateral to the medial physis of the clavicle and did not involve the sternoclavicular joint. Internal fixation was achieved in an anatomic position with an elastic intramedullary nail. The postoperative course was unremarkable and resulted in complete healing of the fracture in an anatomic position. The patient returned to full activities without any pain or dysfunction.

**Conclusion:**

The use of elastic intramedullary nails is a viable option for internal fixation of displaced medial clavicle fractures. Knowledge of the surgical anatomy, potential implant complications, and rehabilitation principles is essential to a successful outcome.

## 1. Introduction

The least common type of clavicle fracture occurs at its medial end. In the pediatric population, medial clavicle injuries often involve the physis. The medial epiphysis of the clavicle does not typically fuse to the rest of the clavicle until approximately 20 years of age. For that reason, an important distinction to make in pediatric patients is between physeal separation and a true fracture of the medial clavicle. In this case, the injury was a fracture of the medial clavicle with an intact physis. Multiple factors guide the development of an appropriate treatment strategy for medial clavicle fractures in pediatric patients, such as the amount of displacement, potential impact of the fracture on upper extremity function, neurovascular status, skeletal maturity, and activity level of the patient.

## 2. Case Presentation

### 2.1. History

A healthy thirteen-year-old male, who is a competitive basketball and baseball player, sustained a closed, completely displaced fracture involving the medial clavicle after falling off an all-terrain vehicle. He was initially seen in the emergency department, placed in a sling, and referred to the orthopedic service for definitive management. The patient is right-hand dominant and does not smoke. He has no prior history of injury to the right clavicle. At the time of his orthopedic consultation, his pain was described as moderate and sharp localized to the medial end of the right clavicle. The patient reported no numbness or tingling in his arm.

### 2.2. Physical Examination

Physical examination revealed a healthy-appearing, pleasant male responding appropriately and in no apparent distress. The pertinent findings on examination included no signs of cervical radiculopathy, no pain or winging of the scapula, intact skin, but significant tenting and swelling over the medial right clavicle, and obvious asymmetry of the right clavicle compared to the left clavicle consistent with a displaced fracture. The end of the clavicle lateral to the fracture site was displaced anterior to the medial component. The remainder of the physical examination revealed no additional injury to the upper extremity. Motor function and sensation were intact throughout the right upper extremity. The brachial and radial pulses were normal and symmetric to the left upper extremity.

### 2.3. Imaging Studies

**Figure 1:**
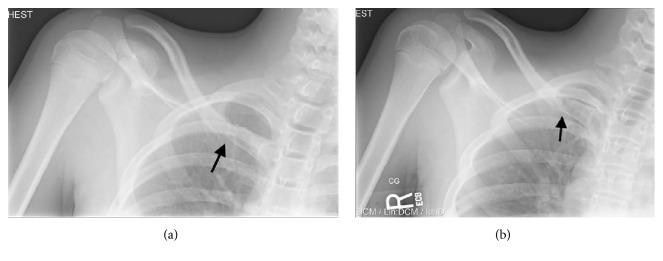
(a) Preoperative AP view and (b) AP cephalic tilt view of the right medial clavicle. The black arrow indicates the site of fracture.

Initial radiographs of the right clavicle revealed a fracture involving its medial end without disruption of the sternoclavicular joint (Figures 1(a) and 1(b)). Subsequent CT scan revealed a completely displaced fracture of the right medial clavicle. The fracture site was lateral to the medial physis of the clavicle and without disruption of the sternoclavicular joint. The CT scan allowed for a more definitive characterization of the fracture pattern. Given the rarity with which medial clavicle fractures not involving the physis occur in the pediatric population, a CT scan was desired to provide this information and to help with surgical planning.

### 2.4. Treatment

Nonoperative and operative treatment options were discussed with the patient and his parents. The factors relevant to pursuing operative treatment included complete displacement at the site of the fracture, desire to obtain anatomic alignment of the fracture to promote healing, potential impact of the displaced clavicle fracture on shoulder function relative to daily activities and overhead sports in basketball and baseball, and clinical outcome allowing the patient to return to activity without pain and restricted function. The operative treatment agreed upon was reduction of the fracture and placement of an elastic intramedullary clavicle nail. Informed consent was signed by the parents and the patient.

**Figure 2:**
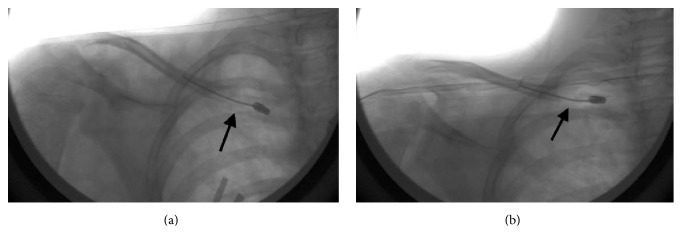
(a) Intraoperative AP view and (b) AP cephalic tilt view of the right medial clavicle. The black arrow indicates the site of fracture.

At the time of the surgery, a trial of closed reduction revealed an unstable fracture. Subsequently, an incision was made centered over the fracture site. Exposure of the fracture site confirmed complete displacement with the lateral end of the clavicle anterior to the medial end. A drill hole using a 2.7 mm drill was made approximately 1 cm lateral to the medial end of the clavicle. The fracture was reduced and then a 1.5 mm elastic intramedullary titanium nail was passed through the drill hole in the metaphyseal area of the clavicle. The nail was advanced into the intramedullary canal, across the fracture site, and into the lateral end of the clavicle. Placement of the intramedullary nail was confirmed under fluoroscopy (Figures 2(a) and 2(b)). The reduction and fixation were stable as the right upper extremity was brought through a full range of motion. An end cap was placed on the medial end of the clavicle nail to prevent migration. After wound closure and placement of a sterile dressing, the right upper extremity was placed in a shoulder immobilizer. The patient recovered from anesthesia without complications. Preoperative and postoperative examinations of motor function, sensation, and pulses were equivalent.

**Figure 3:**
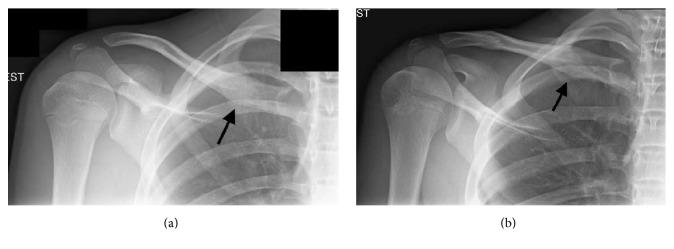
(a) Postoperative AP view and (b) AP cephalic tilt view of the right medial clavicle. The black arrow indicates the site of fracture.

Postoperatively, the patient was immobilized for 6 weeks until healing was evident on radiographs. He then started active-assisted shoulder stretching exercises at home. Although earlier mobilization could have been considered for this patient, we thought it prudent given his age and nature of the fracture to protect him during the immediate postoperative period for 6 weeks. Given the desire to return the patient to his basketball season and knowing the stress he would put on it during other sports requiring overhead activity of his operative arm, we wanted to ensure complete healing and avoid potential mechanical complications relating to the nail during the early postoperative period. At 8 weeks after surgery, the patient started using his right upper extremity for simple activities of daily living and strengthening exercises. At 10 weeks after surgery, he returned to basketball without any pain or functional limitations. Radiographic images in orthogonal planes confirmed complete healing of the fracture (Figures 3(a) and 3(b)). Approximately 18 weeks after his initial surgery, the patient underwent an uncomplicated procedure to remove the intramedullary nail. Two weeks after intramedullary nail removal, the patient returned to activities without complications.

## 3. Discussion

To date, no report has appeared in the peer-reviewed orthopedic literature describing the use of an elastic intramedullary nail for the treatment of a completely displaced fracture of the medial clavicle in a pediatric patient. This case report describes the use of this device as a viable option that can provide anatomic stabilization for healing.

In children, the clavicle is the most commonly fractured bone accounting for 10–15% of all childhood fractures. However, medial clavicle fractures represent only 1–5% of all clavicle fractures in children [1–3]. Due to the exceptional remodeling capabilities of bone in adolescents, clavicle fractures have traditionally been treated nonoperatively. However, nonoperative treatment is not without risk for the development of malunion or nonunion. These complications are rare in children, but should they develop it would potentially impact shoulder and scapulothoracic function, especially in an athlete requiring above shoulder use of their arm [4].

Displaced clavicle fractures can result in shortening of the clavicle and can alter shoulder posture, scapular rhythm during motion, and biomechanical function of the glenohumeral joint. In athletes, who participate in sports requiring use of their arm above shoulder level, these changes are of primary concern due to the impact they can have on the performance and on the development of injury (e.g., impingement, rotator cuff pathology, and labral tears) [5]. Therefore, anatomic reduction and fixation of the completely displaced clavicle fracture in this case was a primary objective of treatment.

Reported cases of pediatric clavicle fractures treated operatively most commonly involve the middle clavicle. Surgical treatment of the medial clavicle in pediatric patients has been described, but in the context of injury to the physis and/or sternoclavicular joint [1–3, 5–10]. In contrast, this case report of a pediatric patient describes the surgical treatment of a completely displaced medial clavicle fracture lateral to the physis and with an intact sternoclavicular joint.

Multiple forms of surgical management have been described in regard to clavicle fracture fixation including cerclage wires, Kirschner wires, nonabsorbable suture, and plates. Recently, elastic intramedullary nails have become a more common treatment option for pediatric clavicle fractures [1]. The advantages of elastic intramedullary nails include a smaller incision, minimal periosteal stripping and soft tissue dissection, ease of removal, and a decreased risk of migration when compared to Kirschner wires. However, complications can occur and include skin irritation from the prominent medial end of the nail, migration, and breakage of the nail [11, 12]. Additionally, surgeons need to be aware of the precarious anatomy surrounding the medial clavicle. Vital structures posterior to this area need to be protected throughout the procedure to avoid catastrophic complications. In the literature, these benefits and complications have only been described for midshaft clavicle fractures. The data is lacking for medial clavicle fractures, especially in the pediatric population.

Nevertheless, in this case report, we describe the use of an elastic intramedullary nail as a viable surgical technique to reestablish anatomic alignment for appropriate healing and restoration of normal function. Critical to the successful use of the elastic intramedullary nail for pediatric medial clavicle fractures are knowledge of the surgical anatomy, limitations of the nail, and appropriate rehabilitation principles. Criteria used for returning athletes to sports after this surgery include asymptomatic healing of the fracture, radiographic evidence of healing in orthogonal planes, and achievement of rehabilitation goals with regard to range of motion and strength.

## 4. Conclusion

This is an original case report describing the use of an elastic intramedullary nail for the treatment of a completely displaced fracture of the medial clavicle in a pediatric patient. Surgery for this type of fracture can be performed using an elastic intramedullary nail and results in anatomic healing and return to function. Further research is needed to confirm the efficacy of this fixation method in comparison to other surgical options.
